# Distinctions and associations between the microbiota of saliva and supragingival plaque of permanent and deciduous teeth

**DOI:** 10.1371/journal.pone.0200337

**Published:** 2018-07-06

**Authors:** Weihua Shi, Jing Tian, He Xu, Qiong Zhou, Man Qin

**Affiliations:** Department of Pediatric Dentistry, Peking University School and Hospital of Stomatology, Beijing, China; Kyushu Institute of Technology, JAPAN

## Abstract

**Background:**

Using salivary microbiota as an accurate proxy for monitoring supragingival microbiota remains controversial because their relationship remains unclear. The eruption of permanent teeth and the exfoliation of primary teeth in mixed dentition greatly alter microbial habitats, which may cause compositional shifts of oral microbiota from childhood to adults.

**Objective:**

This study’s purpose was to assess whether saliva represents a suitable sample for monitoring supragingival microbiota in healthy people, and to explore how the replacement process of deciduous teeth with permanent teeth in mixed dentition influences microbiota within the oral cavity.

**Design:**

Samples of saliva and of supragingival plaque from permanent and deciduous teeth were collected separately from 20 healthy children with mixed dentition. To characterize their microbial communities, we used the V3–V4 hypervariable region of the bacterial 16S rRNA gene sequence.

**Results:**

Saliva harbored a less even and less diverse community than did the plaque. Discriminating genera, namely *Rothia* and *Streptococcus*, contributed to the saliva and plaque differentiation. About half of predicted KEGG pathways varied between the plaque and saliva communities. Oral bacteria showed significantly associations between their supragingival and salivary states. We identified 20 supragingival plaque-related genera in saliva, such as *Corynebacterium*, *Capnocytophaga*, *Fusobacterium*, and *Neisseria*. Additionally, the relative abundance of Actinobacteria peaked in the permanent teeth plaque but subsided in deciduous teeth plaque and saliva. The exfoliation of deciduous teeth and eruption of permanent teeth might be related to the reported fluctuation in the relative abundance of Actinobacteria from primary dentition to permanent dentition within the oral cavity. The variation between PT and DT was due mainly to permanent teeth being enriched in *Actinomyces* and deciduous teeth in *Treponema*.

**Conclusion:**

These results suggested that the supragingival plaque-related bacteria could be suitable candidates when sampling saliva for monitoring supragingival microbiota. The replacement process of deciduous teeth with permanent teeth in mixed dentition might be related to the reported age-maturation of phylum Actinobacteria in the oral cavity.

## Introduction

Although both saliva and supragingival plaque are commonly sampled in oral microbial studies, the relationship between salivary and supragingival microbiota remains inadequately known. There are considerable differences in microbial diversity and composition between saliva and supragingival plaque [1, 2]. Since the principal niche of cariogenic bacteria is supragingival plaque, some researchers have argued that using salivary microbiota as a proxy for supragingival microbiota may not provide meaningful conclusions in dental health and caries studies [3, 4]. Yet the fact that supragingival environment is constantly bathed by saliva and saliva contains bacteria that are shed from tooth surfaces suggests the possibility of using salivary microbiota for monitoring supragingival microbiota. Several components of the salivary microbial populations are reportedly linked to different dental conditions [5, 6]. Obtaining a saliva sample is non-invasive, and it is simple to collect and store, safe to handle, cost-effective, and contain high-quality DNA [7, 8]. These sampling advantages also make saliva a promising specimen in oral investigations, especially for children who are unable to cooperate with plaque collection. Hence, it is necessary to explore the relationship, especially the association between the microbiota of saliva and supragingival plaque in order to assess the accuracy of using saliva as ‘stand-in’ for dental plaque.

It has been well documented that oral cavity contains dynamic microbial communities throughout human life [1, 9]. The age-driven maturation of oral microbiota is related to several factors such as teeth eruption, changes in dietary habits, hormones and the immune system [10]. For example, hormonal changes may be related to the increase of the frequency of detecting Bacteriodes in subgingival plaque during puberty phase [11]. The eruption of permanent teeth and the exfoliation of primary teeth in mixed dentition greatly alter microbial habitats, which may contribute to fluctuations of supragingival microbiota from childhood to adults [1]. Since the interaction between salivary and supragingival microbiota, the replacement process may also cause the composition shifts of salivary microbiota. Detecting the relationship of microbiota among saliva, permanent teeth plaque, and deciduous teeth plaque could help obtain a better understanding of how the replacement of primary teeth with permanent teeth influences microbial maturation within oral cavity.

A distinguishing feature of mixed dentition is the coexistence of permanent teeth and deciduous teeth, which provide an ideal model for synchronously studying the permanent teeth and deciduous teeth at the same oral cavity. Our previous study demonstrated that permanent teeth harbored distinct microbial profiles from deciduous teeth [12]. More precise sampling is necessary for the proper determination of oral microbial composition and diversity in mixed dentition. Most early studies of microbiota in mixed-dentition focused on saliva alone [13, 14] or the differences between it and pooled supragingival plaque [1]. Further research is needed to detect the relationship of microbiota among saliva, permanent teeth plaque, and deciduous teeth plaque, to obtain a cleared understanding of oral microbiota.

This study’s purpose was to assess whether saliva represents a suitable sample for monitoring supragingival microbiota in healthy people, and to explore how the replacement process of deciduous teeth with permanent teeth in mixed dentition influences microbiota within the oral cavity. We did this by investigating the microbial characteristics and their relations between saliva and plaque from permanent and deciduous teeth in healthy mixed-dentition-stage children.

## Materials and methods

### Ethics statement

All recruitment and study procedures were approved by the Biomedical Ethics Committee of the Peking University School and Hospital of Stomatology (PKUSSIRB-201519003). Written informed consent was obtained from the parents or guardians of all subjects used in this study.

### Study population and sample collection

The subjects consisted of 20 mixed-dentition-stage students, aged 78–123 months, with an eruption of 10–12 permanent teeth at the Huarun Primary School and Zhentai Primary School in Shaoshan (Hunan, China). They were medically healthy, free of systemic and oral diseases (S1 Table). They were the same sample population investigated in our prior study, which details the clinical examination, the inclusion and exclusion criteria [12].

Supragingival plaque of the first permanent molars (PM), deciduous molars (DM), deciduous canines and incisors (DC) and permanent incisors (PI) and unstimulated saliva were separately collected. The sampling site of supragingival plaque was insolated with cotton rolls. Then, each supragingival plaque specimen was pooled from the smooth surfaces of all corresponding teeth by using an individual sterile dental excavator. Plaque samples were immediately released from the excavator by agitation into a sterile, labeled 1.5-mL sterile centrifuge tube (Axygen, USA) containing 1 mL TE buffer (10 mM Tris-HCl, 1 mM EDTA; pH 8.0), which was put on ice. Each subject was instructed to expectorate into a sterile 50-mL centrifuge tube to collected approximately 3 mL of saliva. Saliva samples were collected within 5 min and immediately put on ice. Specimens were transported to local laboratory within 2 h and stored at –20°C. All samples were obtained within 5 days, brought on dry ice to the microbiology laboratory at the Peking University School of Stomatology (Beijing) within 10 h, and frozen at –80°C before isolating their DNA.

### DNA extraction and 16S-rRNA gene amplicon sequencing

In the laboratory, the total bacterial genomic DNA was extracted from all the samples by using the QIAamp DNA micro Kit (Qiagen, Germany), following the manufacturer’s instructions, after an initial treatment with lysozyme (20 mg/ml, 37°C for 1 h) [15]. The quantity and quality of the extracted DNA were respectively measured by agarose gel electrophoresis and a NanoDrop spectrophotometer (Thermo Fisher Scientific, USA). All DNA samples were stored at –20°C until further processing.

Sequencing of the 16S rRNA gene amplicon was done by Beijing Auwigene Tech, Ltd (Beijing, China). The V3-V4 region of the 16S rRNA gene was amplified by PCR (95°C for 5 min; followed by 25 cycles of denaturation 95°C for 30 s, annealing at 56°C for 30 s, extension at 72°C for 40 s; and a final extension at 72°C for 10 min). The PCR primers used were 338F (5’-GTACTCCTACGGGAGGCAGCA-3’) and 806R (5’-GTGGACTACHVGGGTWTCTAAT-3’) [16], which incorporated sample barcode sequences. The PCR amplicons were separated by 1%-agarose gel electrophoresis and a c. 460-bp fragments were purified with an Agencourt AMPure XP kit (Beckman Coulter, Inc., USA). The sequencing was performed on a Illumina Miseq PE300 platform (Illumina, Inc., USA) followed the vendor’s standard protocols.

### Sequencing data analysis

We have investigated the microbial profiles of different sites within the dentition [12]. In the present study, we merged sequencing data of PM and PI as supragingival plaque of permanent teeth, and of DM and DC as supragingival plaque of deciduous teeth. Finally, three separate samples from each subject (60 in total) were analyzed: supragingival plaque of permanent teeth, supragingival plaque of deciduous teeth, and unstimulated saliva (their microbiota were denoted as PT, DT, and S, respectively).

The QIIME pipeline (v1.9.1) [17] was used to process the data generated. In brief, raw sequences with exact matches to the barcodes were assigned to each sample, and the primers and barcodes were trimmed for further quality control. Sequences < 200 bp or with average quality scores < 20, including ambiguous base calls (N), were removed. Chimeras were identified and removed by the UCHIME algorithm [18]. Because of differences in the number of sequences per sample, bacterial diversity information from these data was not comparable [1]. All 16S libraries were randomly subsampled to 15,000 sequences per library for the downstream analyses. Sequences were clustered into operational taxonomic units (OTUs) at a 97% similarity cutoff, by using the de novo OTU picking strategy (pick_de_novo_otus.py, uclust) in QIIME v1.9.1 [17]. The Greengenes (13_8) Database was used to assign sequences to specific microbial taxonomy. Singleton OTUs were removed before any further analyses.

Beta diversity metrics were generated and plotted following a principal coordinates analysis (PCoA), and compared between and within groups by the Kruskal—Wallis test. The Dunn-Bonferroni method was performed for post hoc testing after a significant Kruskal—Wallis test using IBM SPSS Statistics (version 20). Alpha diversity was estimated as microbial richness (Observed OTUs), evenness (Equitability index), and diversity (Shannon index). Relative abundances of microbial taxa at the phylum, class, order, family and genus levels were calculated and compared. Alpha diversity metrics and differences in the relative abundances of taxa and KEGG pathways across three sample types of the same individual were analyzed using the Friedman test, a test for multiple related or paired samples. Post hoc analysis was conducted automatically in SPSS Statistics. A random forest algorithm was used to identify the genera responsible for the differential distributions between the supragingival plaque and saliva communities. The ‘randomForest’ package for R (v3.4.0) [19] was used with its default settings. To explore the supragingival plaque-associated genera occurring in saliva, we used the Spearman correlation coefficient (SCC) values for the relative abundances of each genus between PT and S (PT-S), and between DT and S (DT-S). Genera with SCC > 0.5 or < –0.5 at *P* < 0.05 are selected and rendered into microbial network module via Java Cytoscape (v3.4.0) [20]. Microbial functions were predicted using PICRUSt (v1.0.0) following the online protocol [21] and aligned to the Kyoto Encyclopedia of Genes and Genomes (KEGG) database. Relative abundances of functional pathways were calculated and compared by Friedman test and the post hoc comparisons using SPSS.

### Data accession

The sequencing data was added to the SRA database under accession number SRP132650.

## Results

### Global sequencing data

After data trimming and quality filtering, a total of 2,550,879 high-quality sequences (representing ~76% of the raw sequences) were generated, with an average of 42,514 sequences per sample (ranging from 17,129 to 67,405; S1 Table). These sequences were clustered into 6234 operational taxonomical units (OTUs), with 488–970 OTUs per individual specimen (S1 Table).

### Microbial community diversity

**Fig 1:**
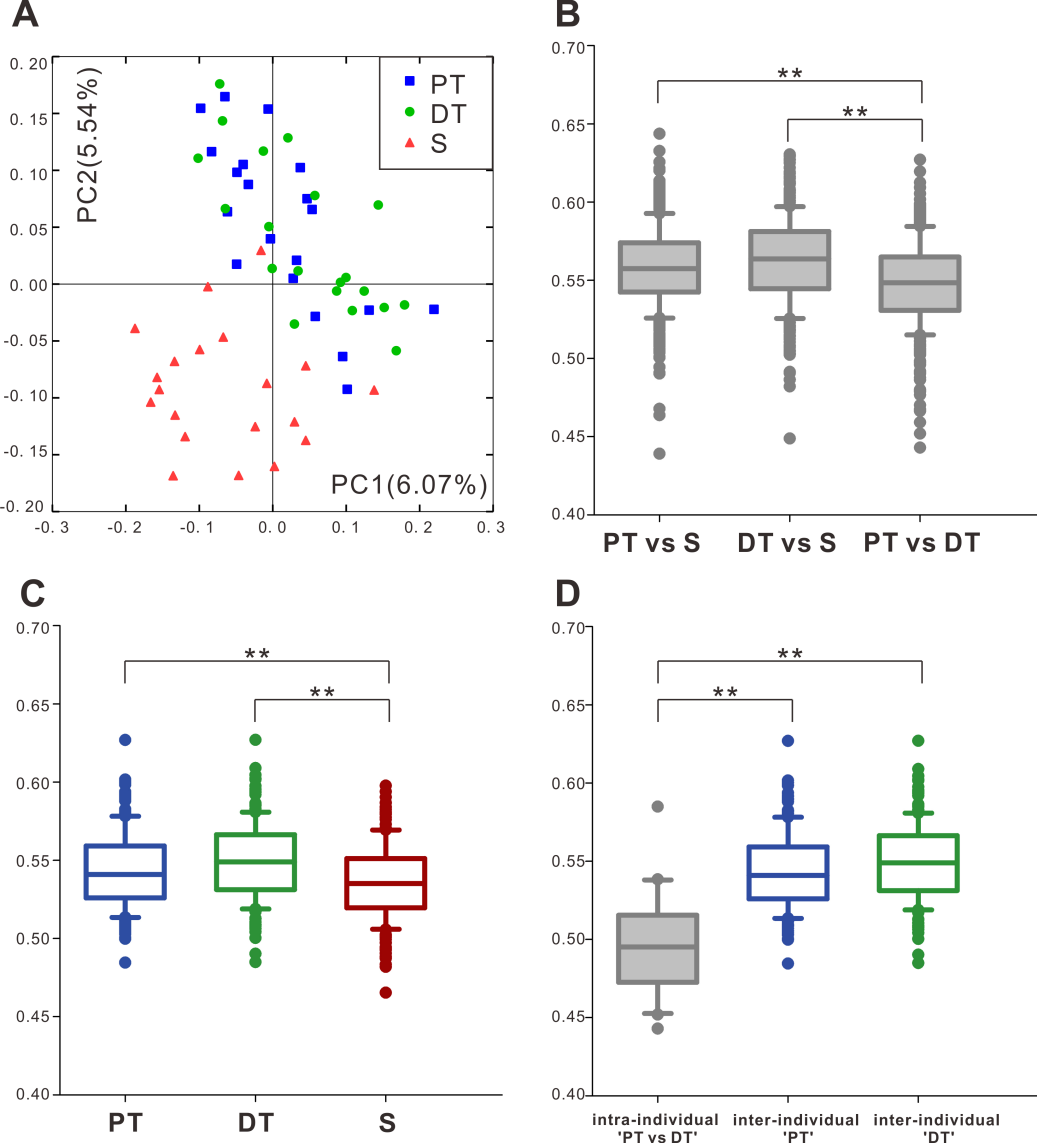
Beta diversity of microbial communities of permanent teeth plaque (PT), deciduous teeth plaque (DT), and saliva (S) based on the unweighted UniFrac distances. (A). Principal coordinate analysis (PCoA). (B). Between-group dissimilarities in the microbial community structures. (C). Within-group dissimilarities of PT, DT, and S. (D). The beta diversities between different sample types from the same child (intra-individual 'PT vs DT') are smaller than those between the same sample types from different child (inter-individual 'PT' and 'DT'. ** *P* < 0.01 by Kruskal—Wallis test and a Dunn-Bonferroni test for post hoc comparisons. In B, C and D, the boxplots show the medians and the 10^th^, 25^th^, 75^th^, and 90^th^ percentiles of each sampled group.

The overall dissimilarities of microbial community structure among the three groups (i.e., beta diversity) were compared by using the unweighted UniFrac distance. A principal coordinates analysis (PCoA) plot revealed that S was distinct from both PT and DT, whereas PT and DT tended to be more similar to each other (Fig 1A). The between-group distances indicated that the degree of variation between PT and S was similar to that observed between DT and S (*P* = 0.093), but both were significantly larger than that found between PT and DT (P < 0.01, Fig 1B). The microbial composition within each group varied least in S and more so in PT and DT (*P* < 0.01, Fig 1C). From the PCoA of Fig 1, PT and DT samples were clustered closely with each other. The beta diversities between different sample types from the same child (intra-individual 'PT vs DT') are smaller than those between the same sample types from different child (inter-individual 'PT' and 'DT', *P* < 0.01, Fig 1D).

**Fig 2:**
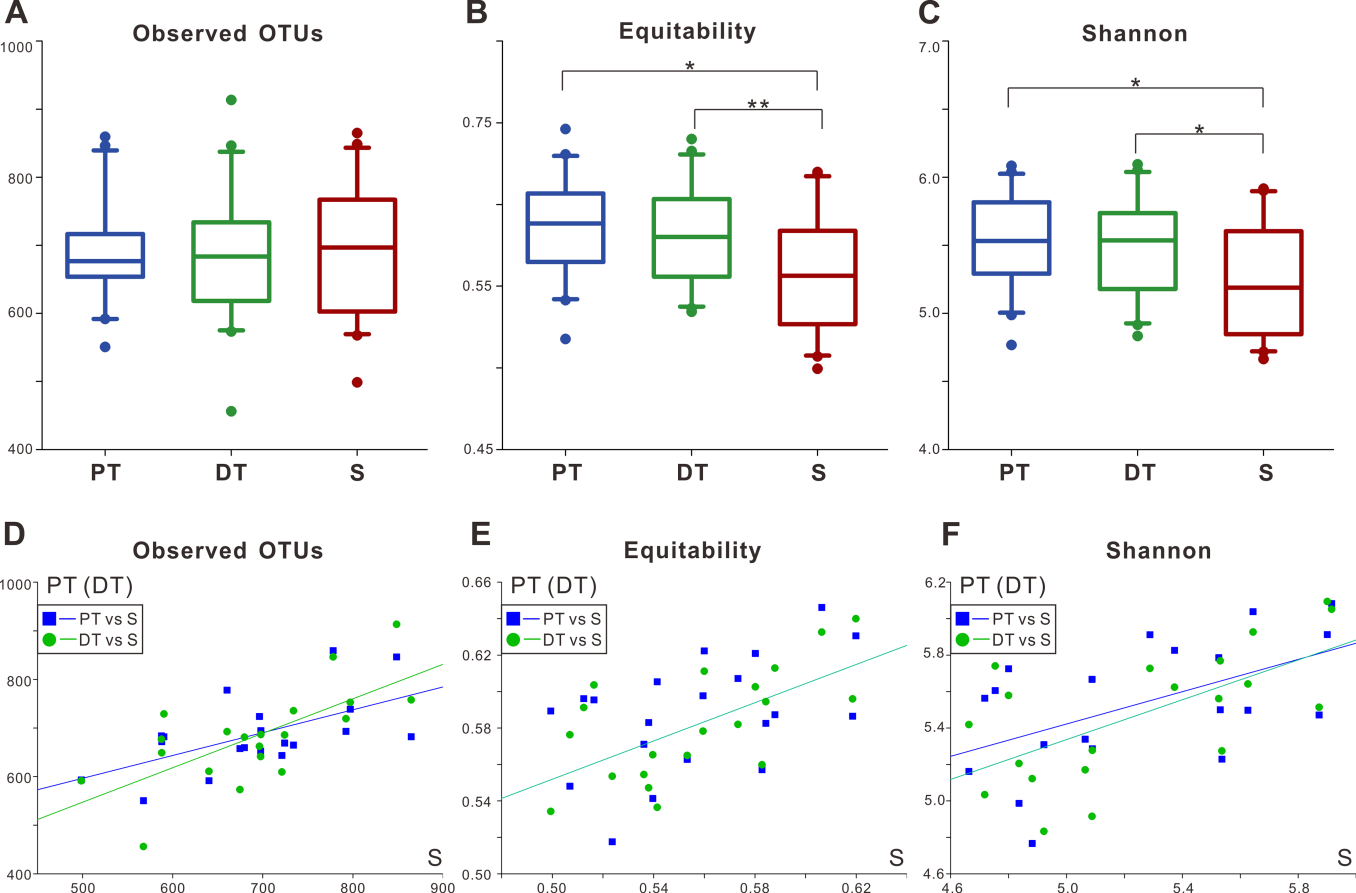
Alpha diversity of the microbial communities in permanent teeth (PT), deciduous teeth (DT), and saliva (S). (A–C). Boxplots of three alpha diversity estimators for the three sampled groups; * *P* < 0.05, ** *P* < 0.01 by the post-hoc analysis for Friedman test in SPSS. (A). The observed OTUs index (richness estimator) was similar among the three groups. (B, C). The equitability (evenness estimator) and Shannon (diversity estimator) indices showed that PT and DT harbored significantly more even and diverse microbial communities than did S. (D–F). Alpha diversity relationships between the plaque groups (including PT and DT) and the S group. Correlation curves represent significant relationships (*P* > 0.05) between PT or DT and S. All indices of PT and DT were significantly correlated with that of S group (Spearman, *P*-values < 0.05), except the equitability index of PT (*P* = 0.081).

Fig 2 showed the microbial diversity within each sample (the alpha diversity). The observed OTUs index showed no significant difference among the groups (*P* = 0.549, Fig 2A). The equitability and Shannon indices demonstrated that the S microbial community was significantly less even and diverse than were the plaque samples from PT and DT by comparing pairwise (*P* < 0.05, Fig 2B and 2C). The inter-group correlations of alpha diversity were also explored (Fig 2D–2F, S2 Table). The three alpha diversity indices were significantly correlated (Spearman, *P*-values < 0.05) among PT, DT, and S, with the exception of the equitability index between PT and S (*P* = 0.081).

### Taxonomic characterization of the 16S rRNA gene profiles

**Fig 3:**
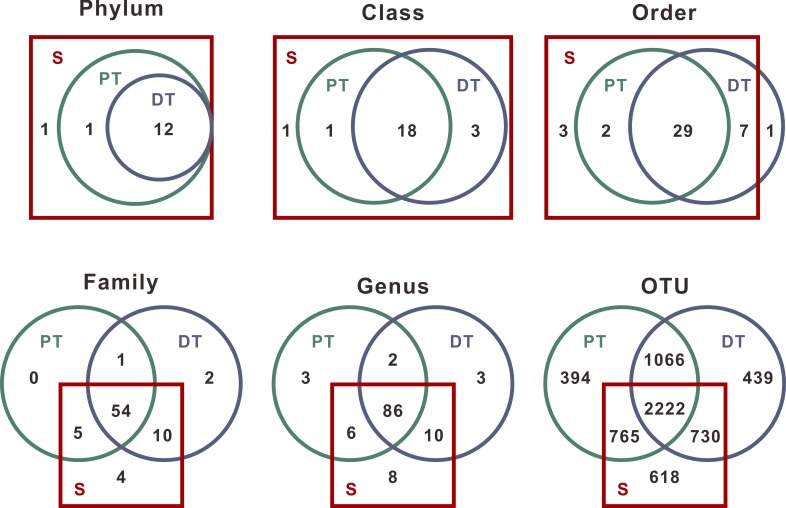
Shared and unique taxa among the three oral cavity niches. Green and blue circles represent the microbial sets of permanent and deciduous teeth plaque samples, respectively. The red rectangle represents the microbial community in saliva. These diagrams depict the relevant relationships of three groups and numbers of taxa contained at different levels. At the phylum and class levels, saliva contained all microbial taxa lodged in the supragingival plaque of permanent and deciduous teeth. At the lower levels, however, the three groups shared most of the detected taxa but also harbored their own.

A total of 14 phyla, 23 classes, 42 orders, 76 families, 118 genera, and 6234 OTUs were detected from the 60 samples. At the phylum and class levels, S contained all microbial taxa detected in PT and DT. At the order, family and genus levels, most taxa (29/42 orders, 54/76 families, and 86/118 genera) were shared among the three groups. Saliva and supragingival plaque also harbored particular taxa unique to them, especially at lower levels (Fig 3).

**Fig 4:**
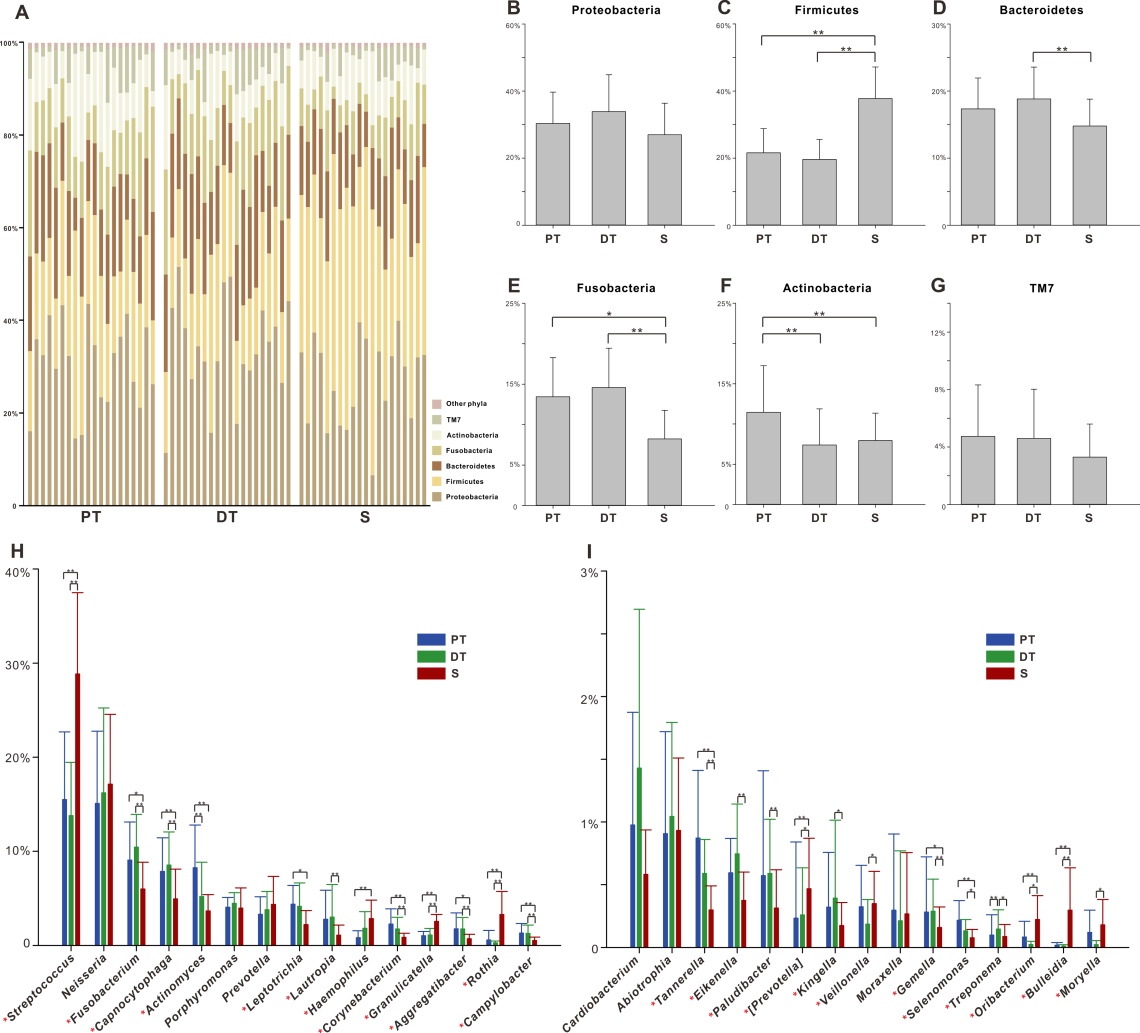
Microbial composition of permanent teeth plaque (PT), deciduous teeth plaque (DT), and saliva (S) at phylum and genus levels. (A). Relative abundances of the resident bacterial phyla in all samples of the three groups. (B–G). Relative abundances of the six main bacterial phyla. (H, I). Genera with a mean relative abundance value > 0.1% are shown; these 30 genera are presented in descending order of mean relative abundances. All bars are the mean ± SD. * *P* < 0.05, ** *P* < 0.01 by the post-hoc analysis for Friedman test in SPSS.

The composition and distribution of microbiota were then characterized in terms of the relative abundances at different taxonomic levels. The six most abundant phyla were Proteobacteria (7–52% of the total sequences), Firmicutes (13–57%), Bacteroidetes (7–29%), Fusobacteria (3–23%), Actinobacteria (2–24%) and TM7 (1–12%). These six phyla were detected in all samples and together accounted for more than 97% of the total sequences (Fig 4A). The mean relative abundances of Firmicutes, Fusobacteria and Actinobacteria showed significant differences between PT and S. Meanwhile, Firmicutes, Bacteroidetes, and Fusobacteria varied between DT and S (Fig 4B–4G). Other relative rare phyla were GN02, Spirochaetes, SR1, Tenericutes, Synergistetes, Cyanobacteria, and Lentisphaerae.

A total of 118 genera were detected. From these, we selected those genera with mean relative abundance values > 0.1% and compared their distribution among the three groups. In contrast to S, PT contained 17 genera that showed significant differences in relative abundance, while DT had 21 such genera (Fig 4H and 4I). *Fusobacterium*, *Capnocytophaga*, *Corynebacterium*, etc. exhibited higher relative abundance in supragingival plaque (PT or DT), whereas *Streptococcus*, *Granulicatella*, *Rothia*, etc. were more abundant in saliva (S3 Table). The variation between PT and DT was due mainly to permanent teeth being enriched in *Actinomyces* and deciduous teeth in *Treponema* (Fig 4H and 4I).

The random forest analysis further validated the distinctions between the supragingival plaque and saliva communities. Genera contributing to the differentiation between PT and S, according to their importance values, included *Rothia*, *Granulicatella*, *Bulleidia*, *Actinobacillus*, *Streptococcus*, *Actinomyces*, *Corynebacterium*, *Mogibacterium*, *Peptostreptococcus*, and *Atopobium*. As to the differentiation between DT and S, the discriminating genera different from those between PT and S, included *Rothia*, *Streptococcus*, *Bulleidia*, *Oribacterium*, *Granulicatella*, *Tannerella*, *Atopobium*, *Mogibacterium*, *Fusobacterium*, and *Peptostreptococcus* (Table 1). The 10 top-ranked important genera all showed significant difference of relative abundance in Friedman test. Genera with significantly different distribution, however, had their own important values and the important values were not positively related to their mean relative abundance. For example, *Streptococcus* was the most abundant genera but only had 5th important values in PT-S.

**Table 1:** The 10 top-ranked important genera contributing to the differentiation between the supragingival plaque and saliva communities.

Microbial communities	Genera	Mean decrease accuracy	Enriched group
PT—S *	*Rothia*	10.02	S
PT—S	*Granulicatella*	9.03	S
PT—S	*Bulleidia*	7.87	S
PT—S	*Actinobacillus*	7.17	S
PT—S	*Streptococcus*	6.99	S
PT—S	*Actinomyces*	6.79	PT
PT—S	*Corynebacterium*	6.71	PT
PT—S	*Mogibacterium*	6.24	S
PT—S	*Peptostreptococcus*	5.63	S
PT—S	*Atopobium*	4.74	S
DT—S **	*Rothia*	12.5	S
DT—S	*Streptococcus*	8.62	S
DT—S	*Bulleidia*	7.75	S
DT—S	*Oribacterium*	7.12	S
DT—S	*Granulicatella*	5.72	S
DT—S	*Tannerella*	5.1	DT
DT—S	*Atopobium*	4.43	S
DT—S	*Mogibacterium*	4.23	S
DT—S	*Fusobacterium*	4.12	DT
DT—S	*Peptostreptococcus*	3.71	S

* PT—S, Random forests analysis of permanent teeth plaque (PT) and saliva (S) communities.

** DT—S, Random forests analysis of the deciduous teeth plaque (DT) and saliva (S) communities. Mean decrease accuracy is an importance value in the ‘randomForest’ package in R.

**Fig 5:**
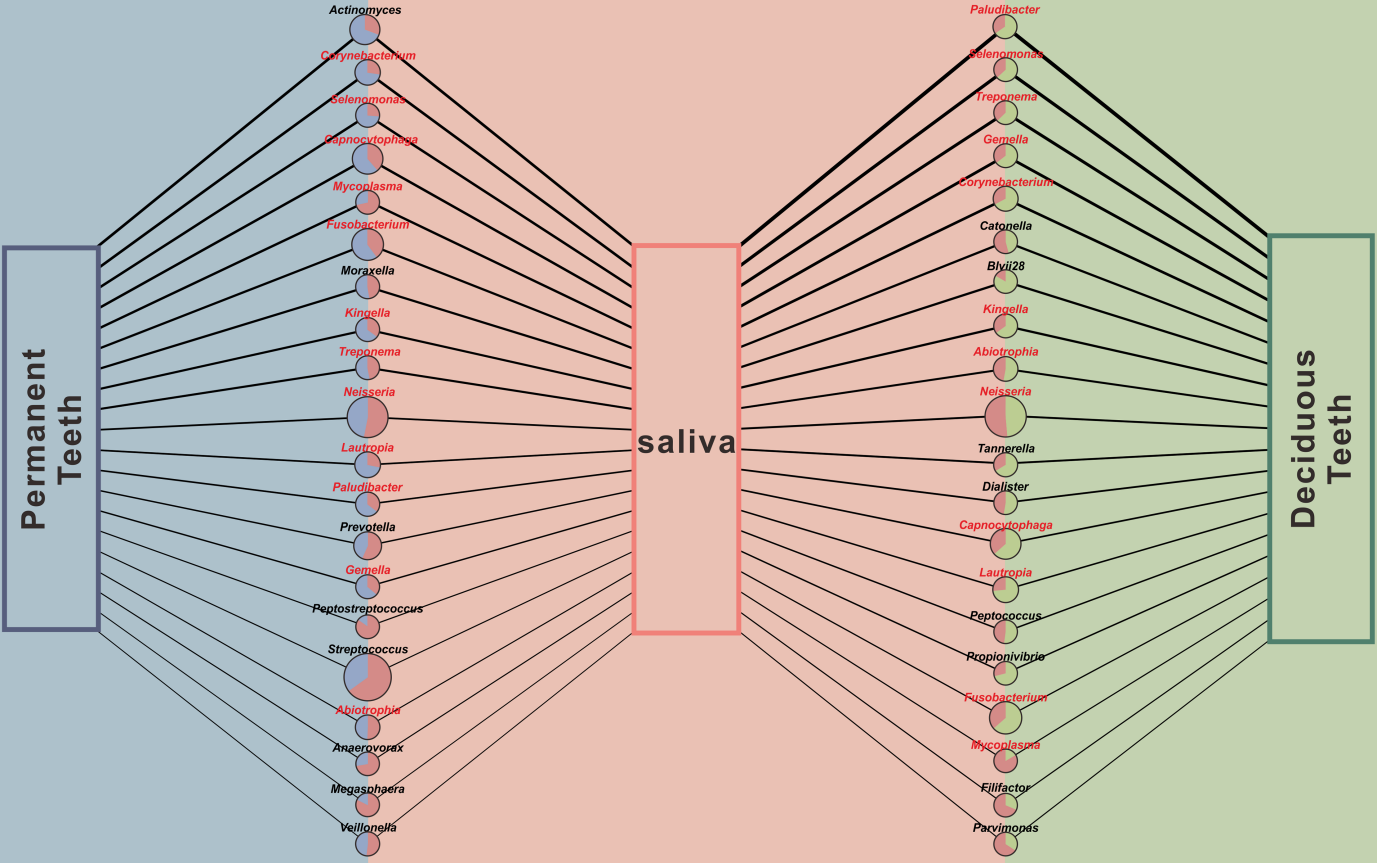
Supragingival plaque-correlated genera in saliva. The Spearman correlation coefficient (SCC) values for the relative abundance between the plaque and saliva groups of each genus were respectively calculated. In the PT-vs.-S and DT-vs.-S analyses, genera with *r*_*s*_ > 0.5 or *r*_*s*_ < –0.5 at *P* < 0.05 are shown as circles arranged in decreasing order of SCC values. The circle size indicates the mean relative abundance in the corresponding plaque (PT or DT) and saliva (S) groups. Each dot is a pie chart consisting of two slices: blue and green slices indicate the relative abundances of the corresponding genus in permanent teeth (PT) and deciduous teeth (DT), respectively, and the pink slice indicates that of saliva (S). All selected genera had positive SCC values, indicated by the lines linking PT or DT with S. Line thickness corresponds to SCC. Red font indicates those genera occurring in both PT-S and DT-S analyses.

After calculating the Spearman correlation coefficient (SCC) values for the relative abundances of each genus between PT and S (PT-S), and between DT and S (DT-S), 44 genera showed positive SCC values and 6 genera showed negative values in PT-S analysis, while 46 genera and 5genera showed positive and negative SCC values respectively in DT-S analysis (S4 Table). We considered any taxon whose relative abundance was significantly strong correlated (r_s_ < -0.5 or r_s_ > 0.5, *P* < 0.05) [22] between its salivary and supragingival plaque states, as being a plaque-correlated taxon in saliva. At the genus level, for both PT-S and DT-S, 20 genera finally fit the criterion and all these genera had positive relationships. These selected genera with were rendered into a network module (Fig 5). High SCC values between PT and S were found for *Actinomyces* (*r*_*s*_ = 0.792), *Corynebacterium* (*r*_*s*_ = 0.783) and *Selenomonas* (*r*_*s*_ = 0.763); between DT and S, *Paludibacter* (*r*_*s*_ = 0.962), *Selenomonas* (*r*_*s*_ = 0.886) and *Treponema* (*r*_*s*_ = 0.847) exhibited the strongest associations (S5 Table).

### Functional content prediction

A total of 4592 KEGG ortholog groups (KOs) were predicted and 3548 KOs (77.26%) were shared among all 60 samples. The number of KOs per sample averaged 3975 ± 79, 4017 ± 63 and 4043 ± 77 in PT, DT, and S, respectively.

At the pathway level, a total of 261 KEGG pathways were predicted among all the profiles. We selected those pathways with mean relative abundance values > 0.1% for downstream analyses. Of the 140 pathways predicted, 69 pathways differed in their relative abundances between PT and S, while 91 pathways differed between DT and S (S6 Table). Interestingly, pathways related to aerobic metabolism, such as citrate cycle (TCA cycle) and oxidative phosphorylation, were more abundant in plaque (PT or DT groups). We also explored the functional characteristics of the supragingival plaque-correlated genera in saliva. As S1 Fig showed, in the PT and S groups, *Streptococcus*, *Kingella*, *Prevotella*, *Megasphaera*, and *Veillonella* had similar correlation patterns for many pathways—for example, all were positive correlated with glycolysis, fructose metabolism, and D-Alanine metabolism, yet negatively so with arginine metabolism—and could be clustered into functional subgroups. In DT and S, *Gemella*, *Paludibacter*, *Treponema*, *Tannerella*, and *Fusobacterium* were clustered because of their similar correlations with cell motility and energy metabolism pathways.

## Discussion

Analysis of the data in this article presented the differences and associations in microbial diversity, composition and functional characters between saliva and supragingival plaque of permanent and deciduous teeth within the same oral cavity in the healthy children with mixed dentition. Simón-Soro et al reported that considerable differences in bacterial composition between teeth at different intra-oral locations. The more pronounced differences were observed between saliva and supragingival plaque than between teeth at different intra-oral locations [4]. To focus on the supragingival profiles of teeth in various locations and not be subject to interference from salivary microbiota, we just processed and analyzed the sequencing data of supragingival plaque samples in our previous study [12]. In the present research, in order to explore the microbial relations between saliva and plaque, we used these supragingival plaque samples and included additional saliva samples of the corresponding subjects.

To assess the accuracy of using salivary microbiota for monitoring supragingival microbiota, we conducted a thorough investigation of the microbial characteristics of saliva and of supragingival plaque from permanent teeth and deciduous teeth.

The Shannon index results indicated that the microbial community of saliva was less alpha-diverse than that of either supragingival plaque (PT and DT, Fig 2C). This finding agreed with prior studies of various age stages of the subjects [1, 23]. The Shannon index is an alpha diversity index that ‘crystallizes richness and evenness into a single feature’ [24]. As Fig 2 illustrated, both permanent and deciduous teeth had more even microbial communities than did saliva, while richness (OTUs) was similar among these three niches. This result suggested that evenness might play a more significant role than richness in generating the alpha-diversity differences between saliva and plaque communities in mixed dentition. Several other studies also reported supragingival plaque being more evenly distributed than saliva [2, 23], likely due to the ability of plaque microbiota to accumulate on the non-shedding tooth surface, which provides them with more time, compared with saliva, to develop into an even and complex community [1, 2].

Former studies reported the differences in the relative abundances of taxa between saliva and supragingival plaque in healthy people of different age stages [1, 23, 25]. In the present study, we focused on the healthy children with mixed dentition. The results in our study that Firmicutes were more abundant in saliva while Fusobacteria and Actinobacteria were more abundant in plaque were consistent with previous observations in preschool students [23] and adults [25]. Our previous study demonstrated distinct microbial profiles between supragingival plaque of permanent and deciduous teeth. Physicochemical factors, the residence time in the oral cavity and the surface structure might be related to these considerable differences [12]. Other factors, such as salivary flow, temperature and edox potential might have influence on oral bacteria as well [26–28]. Here, we focused on the microbial communities of saliva and plaque, and identified a group of 10 important discriminatory taxa contributing to the differentiation between saliva and plaque (Table 1). Some of these discriminatory genera are known to play key roles in dental biofilm formation. For example, *Rothia* was reported as one of the important initiators of cell-cell interactions in early biofilm [29]. *Streptococcus*, another initial colonizer of the tooth surface, has been studied in much detail [30, 31].

Saliva also differs from the supragingival plaque of permanent teeth and deciduous teeth in terms of functional potential. About half of tested KEGG pathways (i.e., 69/140 in the PT-S and 91/140 in the DT-S comparison) varied between the plaque and saliva microbial communities. Compared with saliva, supragingival plaque of permanent and deciduous teeth were both significantly enriched with pathways related to aerobic metabolism, including the citrate cycle and oxidative phosphorylation (S6 Table). Since tooth surface is exposed to an aerobic environment, it is likely that oral bacteria encounter residual amounts of oxygen in the dental biofilm development; thus, supragingival plaque would be expected to have a very active and ongoing oxygen metabolism [32, 33].

The above-mentioned findings indicated that saliva harbored different microbial communities from supragingival plaque of permanent teeth and deciduous teeth in terms of diversities, compositions and functional characters. Nevertheless, our results also demonstrated that salivary microbiota showed positive associations with supragingival microbiota. For example, the alpha diversity of PT and DT were positive correlated with that of S (Fig 2), and S contained all taxa of PT or DT at the phylum and class levels (Fig 3). These associations suggested that it might be reasonable to sample saliva for monitoring supragingival microbiota, for both permanent and deciduous teeth.

Some specific taxa, along with their association with host dental phenotypes, have been referred to as biomarkers in saliva for evaluating dental health [34, 35]. Given the heterogeneity of physical niches in oral cavity, there ought to be extra criteria used when screening salivary profiles of dental health. For example, in our study, nearly 10% of OTUs only occurred in saliva. These taxa should not be used for monitoring supragingival microbiota because of their absence in plaque.

Another important criterion to consider is that the taxon level in saliva should reflect its state in plaque. For example, the salivary concentrations of mutans streptococci were noticed to be significantly correlated with their proportions in plaque [36]. Our results detected 20 genera that presented strong correlations between their salivary state and supragingival state of permanent teeth or deciduous teeth in mixed dentition (Fig 5). These supragingival plaque-correlated genera in saliva could serve as promising candidates when sampling saliva for monitoring supragingival microbiota in healthy people.

The supragingival plaque-correlated genera in saliva had variable correlation coefficient values with functional pathways, suggesting complicated synergistic or antagonistic effects on maintaining dental health (S1 Fig). For example, arginine metabolisms of oral microbiota, whose alkali production inhibits tooth demineralization by neutralizing acids, are one of the fundamental pathways that maintain oral health [37–39]. Koopman et al reported that *Neisseria* increased, while *Streptococcus* decreased, in the microbial community of plaque when grown with an arginine supplement [40]. That finding was consistent with the significantly positive correlation of arginine and proline metabolism pathways with *Neisseria* and their negative correlation with *Streptococcus* in our study.

The predominant phyla of saliva and supragingival plaque in the mixed dentition (Fig 4) were consistent with those of primary dentition [23] and permanent dentition [13]. *Capnocytophaga*, *Corynebacterium*, *Campylobacter* were overrepresented in plaque while *Streptococcus*, *Granulicatella* were more abundant in saliva, similar to previous reports of subjects only with deciduous or permanent teeth [23, 25], also indicating the stability of oral microbial distribution in the replacement process. Our results also provided insight into how the replacement process of deciduous teeth with permanent teeth influenced microbiota within the oral cavity by sampling permanent teeth plaque and deciduous teeth plaque separately. For example, Xu et al. reported that saliva harbored more abundant Actinobacteria than did the pooled supragingival plaque in children with primary dentition. However, this trend reversed in young adults, in that pooled supragingival plaque apparently contained more abundant Actinobacteria than saliva in permanent dentition [1]. According to our results, the relative abundance of Actinobacteria peaked in the permanent teeth plaque but subsided in deciduous teeth plaque and saliva. We speculated that the exfoliation of deciduous teeth and eruption of permanent teeth contributed to the reported age-maturation of phylum Actinobacteria in the oral cavity, which manifested as the relative abundance of Actinobacteria increasing with age in the pooled plaque, and thus, in permanent dentition, finally surpassing its relative abundance in saliva.

Subjects in the present study were the children with mixed dentition and their ability to cooperate with sampling was limited. To sample as simply and quickly as possible, supragingival plaque was collected right after oral examination and then saliva was collected immediately. Saliva is generally considered as a reservoir of oral microbiota and contains microorganisms that come not just from the teeth but from other soft tissues [41]. So whether the sampling order will influence the results needs to be further tested.

## Conclusion

The present study showed that saliva was colonized by a microbial community with less even and alpha-diverse than supragingival plaque of permanent teeth and deciduous teeth in the healthy children with mixed dentition. Several taxa and functional pathways were distinctly distributed between saliva and plaque. However, salivary microbiota also showed positive associations with supragingival microbiota. Our study’s findings suggested that the supragingival plaque-related bacteria could be suitable candidates when sampling saliva for monitoring supragingival microbiota. In addition, the replacement process of deciduous teeth with permanent teeth in mixed dentition might be related to the reported age-maturation of phylum Actinobacteria in the oral cavity.

## Supporting information

S1 Fig Functional characteristics of the supragingival plaque-correlated genera in saliva. Supragingival plaque-correlated genera in the PT-vs.-S (A) and DT-vs.-S (B) analyses were respectively estimated in the two heatmaps. Spearman’s correlation coefficient (SCC) values for the relative abundance between the selected genera and KEGG pathways were calculated and color-coded. Only those KEGG pathways relating to cellular processes and metabolism are included in the heatmaps. The 20 selected genera were ordered based on the result of a hierarchical cluster analysis.
(PDF)

S1 Table Basic information of the 20 subjects and their sequencing results. PT, permanent teeth; DT, deciduous teeth; S, saliva
(PDF)

S2 Table The inter-group correlations of alpha diversity. (PDF)

S3 Table The enrichment of significantly different genera between plaque and saliva. *Genera with mean relative abundance values > 0.1% were selected and compared by a post-hoc analysis for Friedman's test in SPSS. The table only lists genera that showed significant differences in relative abundances between plaque (PT or DT groups) and saliva (S group).
(PDF)

S4 Table Spearman correlation coefficient (SCC) values for the relative abundance between the plaque (PT or DT) and saliva (S) groups at genus level. PT—S, correlation of generic relative abundance between permanent teeth plaque (PT) and saliva (S). DT—S, correlation of generic relative abundance between deciduous teeth plaque (DT) and saliva (S).
(PDF)

S5 Table Spearman correlation coefficient (SCC) values for the relative abundance between the plaque (PT or DT) and saliva (S) groups of supragingival plaque-correlated genera in saliva. *PT—S, correlation of generic relative abundance between permanent teeth plaque (PT) and saliva (S). **DT—S, correlation of generic relative abundance between deciduous teeth plaque (DT) and saliva (S).
(PDF)

S6 Table Differentially abundant KEGG pathways between permanent teeth plaque (PT) and saliva (S), and deciduous teeth plaque (DT) and saliva (S). All pathways with *P*-values < 0.05 as tested by a post-hoc analysis for Friedman's test in SPSS are included in this table.
(XLSX)
