# Supplementary figures and images for: Distinctions and associations between the microbiota of saliva and supragingival plaque of permanent and deciduous teeth
Source: PLoS One. 2018 Jul 6;13(7):e0200337. doi: 10.1371/journal.pone.0200337 (PMC6034885; doi:10.1371/journal.pone.0200337)

A

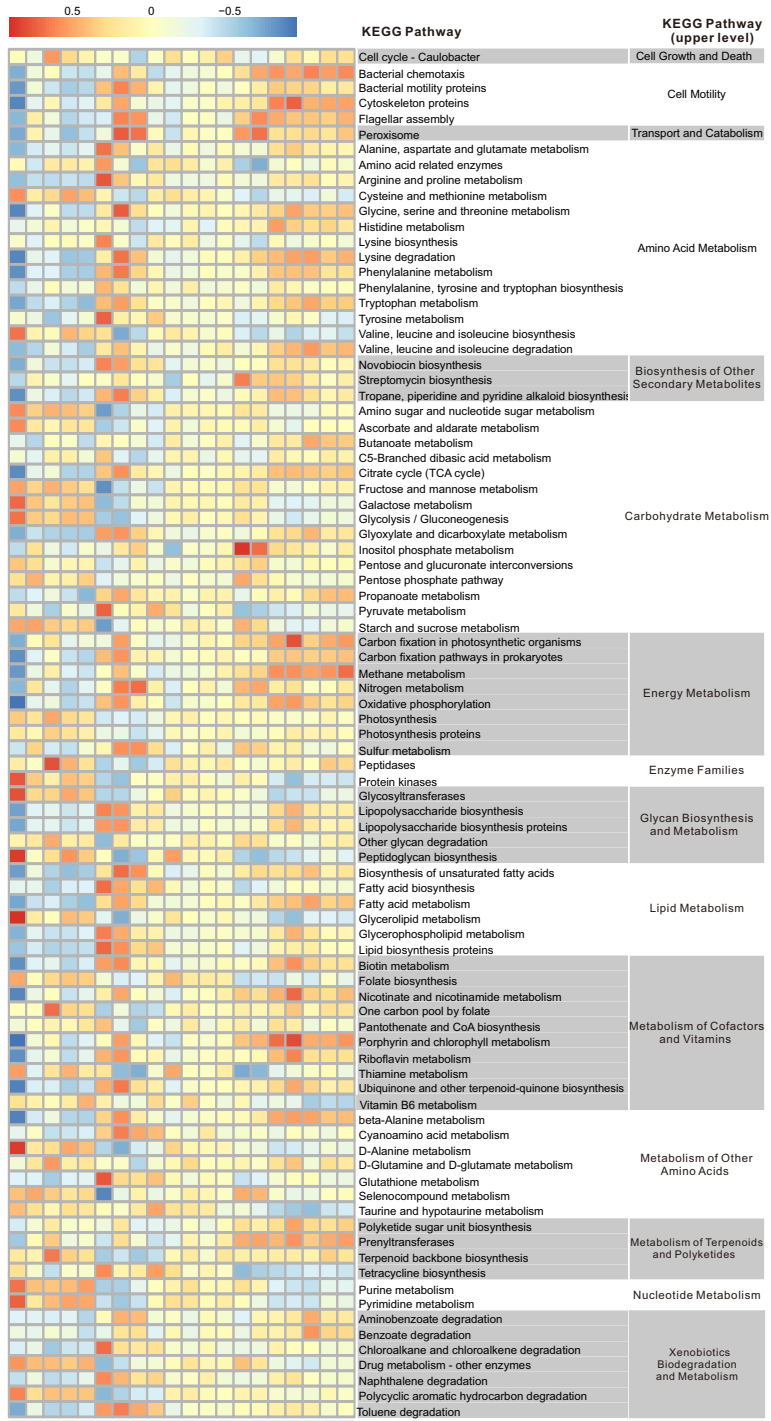

B

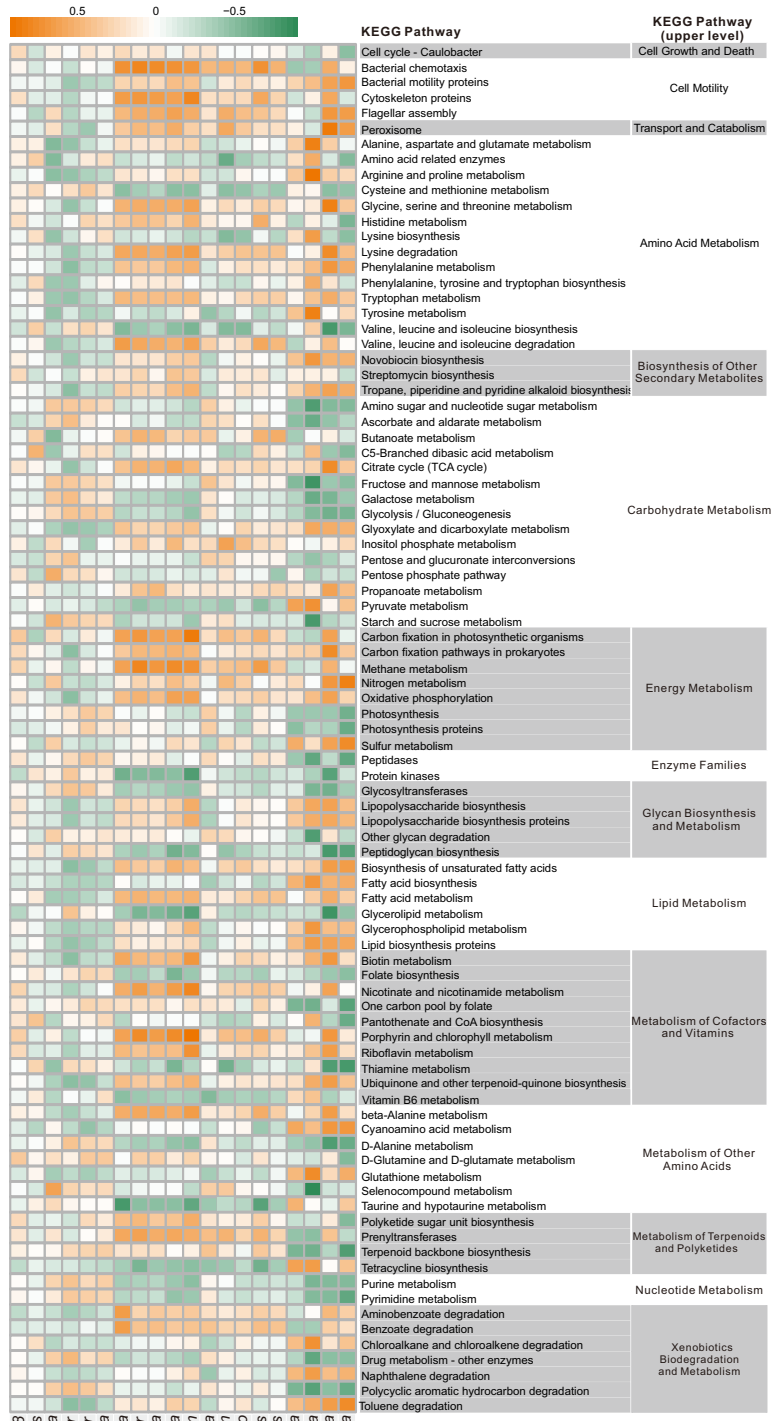

Supplement: S1 Fig — Supragingival plaque-correlated genera in the PT-vs.-S (A) and DT-vs.-S (B) analyses were respectively estimated in the two heatmaps. Spearman’s correlation coefficient (SCC) values for the relative abundance between the selected genera and KEGG pathways were calculated and color-coded. Only those KEGG pathways relating to cellular processes and metabolism are included in the heatmaps. The 20 selected genera were ordered based on the result of a hierarchical cluster analysis. (PDF) [file pone.0200337.s001.pdf]
