# Supplementary material for: Distinctions and associations between the microbiota of saliva and supragingival plaque of permanent and deciduous teeth
Source: PLoS One. 2018 Jul 6;13(7):e0200337. doi: 10.1371/journal.pone.0200337 (PMC6034885; doi:10.1371/journal.pone.0200337)
Supplement: S1 Table — PT, permanent teeth; DT, deciduous teeth; S, saliva (PDF) [file pone.0200337.s002.pdf]

S1 Table.

Subject	Gender (M/F)	Age (months)	Nr of PT	Nr of DT	Sample	Sequences	OTUs
**1**	F	79	10	14	PT	38644	893
					DT	17129	970
					S	19271	901
**2**	F	89	12	12	PT	41966	770
					DT	44409	703
					S	21133	744
**3**	F	94	10	14	PT	45732	700
					DT	58523	611
					S	32638	718
**4**	F	92	10	14	PT	55922	742
					DT	63543	766
					S	25870	848
**5**	M	83	10	14	PT	54021	728
					DT	64510	804
					S	33982	922
**6**	M	78	12	12	PT	42930	727
					DT	26688	724
					S	22756	623
**7**	M	86	10	14	PT	58818	910
					DT	28273	902
					S	31858	823
**8**	M	104	10	14	PT	43637	627
					DT	46566	648
					S	21583	679
**9**	M	105	12	12	PT	41734	713
					DT	47252	691
					S	22454	630
**10**	M	97	10	14	PT	51689	584
					DT	39421	488
					S	25219	604
**11**	F	98	10	14	PT	52765	744
					DT	55681	732
					S	26630	740
**12**	F	93	10	14	PT	64713	724
					DT	67405	776
					S	21494	626
**13**	F	86	12	12	PT	62337	708
					DT	58566	785
					S	31355	783

					PT	56316	685
**14**	M	96	10	14	DT	58611	651
					S	26800	765
					PT	53903	701
**15**	M	94	10	14	DT	55954	724
					S	29565	721
					PT	51946	631
**16**	M	90	12	12	DT	51689	630
					S	19611	532
					PT	49636	696
**17**	M	95	10	14	DT	46209	686
					S	31563	736
					PT	59691	714
**18**	M	92	11	12	DT	55348	735
					S	30567	768
					PT	54812	789
**19**	F	123	12	12	DT	58717	804
					S	27952	850
					PT	58737	827
**20**	F	85	10	14	DT	29318	736
					S	34817	699
