# Supplementary material for: Distinctions and associations between the microbiota of saliva and supragingival plaque of permanent and deciduous teeth
Source: PLoS One. 2018 Jul 6;13(7):e0200337. doi: 10.1371/journal.pone.0200337 (PMC6034885; doi:10.1371/journal.pone.0200337)
Supplement: S2 Table — (PDF) [file pone.0200337.s003.pdf]

S2 Table.

Alpha diversity index	Group	Spearman correlation	
		r_s	*P*
Observed OTUs	PT - S	0.513	0.021
	DT - S	0.723	< 0.001
	PT - DT	0.789	< 0.001
Equitability	PT - S	0.400	0.081
	DT - S	0.626	0.003
	PT - DT	0.568	0.009
Shannon	PT - S	0.481	0.032
	DT - S	0.574	0.008
	PT - DT	0.680	0.001
