# Supplementary material for: Distinctions and associations between the microbiota of saliva and supragingival plaque of permanent and deciduous teeth
Source: PLoS One. 2018 Jul 6;13(7):e0200337. doi: 10.1371/journal.pone.0200337 (PMC6034885; doi:10.1371/journal.pone.0200337)
Supplement: S3 Table — *Genera with mean relative abundance values > 0.1% were selected and compared by a post-hoc analysis for Friedman's test in SPSS. The table only lists genera that showed significant differences in relative abundances between plaque (PT or DT groups) and saliva (S group). (PDF) [file pone.0200337.s004.pdf]

S3 Table.

	**PT vs. S**	**DT vs. S**
**Genera enriched in plaque*** (Genera with a significantly higher abundance in PT or DT groups than in the S group)	*Fusobacterium, Capnocytophaga, Actinomyces, Leptotrichia, Corynebacterium, Aggregatibacter, Campylobacter, Tannerella, Gemella, Selenomonas*	*Fusobacterium, Capnocytophaga, Lautropia, Corynebacterium, Aggregatibacter, Campylobacter, Tannerella, Eikenella, Paludibacter, Kingella, Gemella, Selenomonas, Treponema*
**Genera enriched in saliva*** (Genera with a significantly higher abundance in the S group than in either the PT or DT groups)	*Streptococcus, Haemophilus, Granulicatella, Rothia, [Prevotella], Oribacterium, Bulleidia*	*Streptococcus, Granulicatella, Rothia, [Prevotella], Veillonella, Oribacterium, Bulleidia, Moryella*
