# Supplementary material for: Distinctions and associations between the microbiota of saliva and supragingival plaque of permanent and deciduous teeth
Source: PLoS One. 2018 Jul 6;13(7):e0200337. doi: 10.1371/journal.pone.0200337 (PMC6034885; doi:10.1371/journal.pone.0200337)
Supplement: S4 Table — PT—S, correlation of generic relative abundance between permanent teeth plaque (PT) and saliva (S). DT—S, correlation of generic relative abundance between deciduous teeth plaque (DT) and saliva (S). (PDF) [file pone.0200337.s005.pdf]

S4 Table

Groups	Genera	r_s	*P*
PT - S	*[Prevotella]*	0.232	0.326
PT - S	*Abiotrophia*	0.531	0.016
PT - S	*Acidithiobacillus*	-0.016	0.948
PT - S	*Actinobacillus*	0.192	0.417
PT - S	*Actinomyces*	0.792	0.000
PT - S	*Aggregatibacter*	0.220	0.352
PT - S	*Anaerovorax*	0.530	0.016
PT - S	*Atopobium*	0.206	0.384
PT - S	*Bacillus*	-0.111	0.642
PT - S	*Bulleidia*	-0.179	0.451
PT - S	*Butyrivibrio*	0.047	0.843
PT - S	*Campylobacter*	0.290	0.214
PT - S	*Capnocytophaga*	0.761	0.000
PT - S	*Cardiobacterium*	0.483	0.031
PT - S	*Catonella*	0.484	0.031
PT - S	*Corynebacterium*	0.783	0.000
PT - S	*Dialister*	0.424	0.062
PT - S	*Eikenella*	0.438	0.054
PT - S	*Enterococcus*	0.192	0.417
PT - S	*Filifactor*	-0.180	0.449
PT - S	*Fusobacterium*	0.722	0.000
PT - S	*Gemella*	0.586	0.007
PT - S	*Granulicatella*	0.371	0.107
PT - S	*Haemophilus*	0.006	0.980
PT - S	*Kingella*	0.705	0.001
PT - S	*Lautropia*	0.629	0.003
PT - S	*Leptotrichia*	0.254	0.280
PT - S	*Megasphaera*	0.507	0.022
PT - S	*Mogibacterium*	-0.159	0.504
PT - S	*Moraxella*	0.720	0.000
PT - S	*Moryella*	0.238	0.313
PT - S	*Mycoplasma*	0.745	0.000
PT - S	*Neisseria*	0.639	0.002
PT - S	*Oribacterium*	0.150	0.527
PT - S	*Paludibacter*	0.614	0.004
PT - S	*Parvimonas*	0.377	0.102
PT - S	*Peptococcus*	0.421	0.065
PT - S	*Peptostreptococcus*	0.542	0.013
PT - S	*Porphyromonas*	0.048	0.840
PT - S	*Prevotella*	0.588	0.006
PT - S	*Propionivibrio*	0.435	0.055
PT - S	*Rothia*	0.208	0.380
PT - S	*Schwartzia*	0.392	0.087
PT - S	*Selenomonas*	0.763	0.000
PT - S	*Staphylococcus*	-0.096	0.687
PT - S	*Streptococcus*	0.532	0.016

PT - S	*Tannerella*	0.314	0.177
PT - S	*TG5*	0.299	0.200
PT - S	*Treponema*	0.697	0.001
PT - S	*Veillonella*	0.505	0.023
DT - S	*[Prevotella]*	0.116	0.627
DT - S	*Abiotrophia*	0.681	0.001
DT - S	*Acidithiobacillus*	-0.365	0.113
DT - S	*Actinobacillus*	0.149	0.530
DT - S	*Actinomyces*	0.346	0.135
DT - S	*Aggregatibacter*	0.248	0.292
DT - S	*Anaerovorax*	0.390	0.089
DT - S	*Atopobium*	0.212	0.370
DT - S	*Blvii28*	0.725	0.000
DT - S	*Bulleidia*	0.212	0.369
DT - S	*Butyrivibrio*	0.265	0.259
DT - S	*Campylobacter*	0.438	0.054
DT - S	*Capnocytophaga*	0.626	0.003
DT - S	*Cardiobacterium*	0.397	0.083
DT - S	*Catonella*	0.725	0.000
DT - S	*Corynebacterium*	0.785	0.000
DT - S	*Dialister*	0.644	0.002
DT - S	*Eikenella*	0.453	0.045
DT - S	*Enterococcus*	-0.139	0.558
DT - S	*Filifactor*	0.543	0.013
DT - S	*Fusobacterium*	0.562	0.010
DT - S	*Gemella*	0.821	0.000
DT - S	*Granulicatella*	0.236	0.316
DT - S	*Haemophilus*	0.257	0.274
DT - S	*Halomonas*	-0.053	0.826
DT - S	*Kingella*	0.723	0.000
DT - S	*Lactobacillus*	0.125	0.598
DT - S	*Lautropia*	0.605	0.005
DT - S	*Leptotrichia*	0.409	0.073
DT - S	*Megasphaera*	0.349	0.131
DT - S	*Mogibacterium*	-0.111	0.642
DT - S	*Moraxella*	0.453	0.045
DT - S	*Moryella*	0.100	0.674
DT - S	*Mycoplasma*	0.544	0.013
DT - S	*Neisseria*	0.669	0.001
DT - S	*Oribacterium*	0.136	0.567
DT - S	*Paludibacter*	0.962	0.000
DT - S	*Parvimonas*	0.528	0.017
DT - S	*Peptococcus*	0.595	0.006
DT - S	*Peptostreptococcus*	0.476	0.034
DT - S	*Porphyromonas*	0.087	0.715
DT - S	*Prevotella*	0.421	0.064
DT - S	*Propionivibrio*	0.592	0.006
DT - S	*Rothia*	0.388	0.091

DT - S	*Schwartzia*	-0.076	0.749
DT - S	*Selenomonas*	0.886	0.000
DT - S	*Streptococcus*	0.471	0.036
DT - S	*Tannerella*	0.666	0.001
DT - S	*TG5*	0.480	0.032
DT - S	*Treponema*	0.847	0.000
DT - S	*Veillonella*	0.420	0.066
