# Supplementary material for: Distinctions and associations between the microbiota of saliva and supragingival plaque of permanent and deciduous teeth
Source: PLoS One. 2018 Jul 6;13(7):e0200337. doi: 10.1371/journal.pone.0200337 (PMC6034885; doi:10.1371/journal.pone.0200337)
Supplement: S5 Table — *PT—S, correlation of generic relative abundance between permanent teeth plaque (PT) and saliva (S). **DT—S, correlation of generic relative abundance between deciduous teeth plaque (DT) and saliva (S). (PDF) [file pone.0200337.s006.pdf]

S5 Table.

Groups	Genera	r_s	*P*	Groups	Genera	r_s	*P*
PT - S*	*Actinomyces*	0.792	< 0.001	DT - S**	*Paludibacter*	0.962	< 0.001
PT - S	*Corynebacterium*	0.783	< 0.001	DT - S	*Selenomonas*	0.886	< 0.001
PT - S	*Selenomonas*	0.763	< 0.001	DT - S	*Treponema*	0.847	< 0.001
PT - S	*Capnocytophaga*	0.761	< 0.001	DT - S	*Gemella*	0.821	< 0.001
PT - S	*Mycoplasma*	0.745	< 0.001	DT - S	*Corynebacterium*	0.785	< 0.001
PT - S	*Fusobacterium*	0.722	< 0.001	DT - S	*Blvii28*	0.725	< 0.001
PT - S	*Moraxella*	0.720	< 0.001	DT - S	*Catonella*	0.725	< 0.001
PT - S	*Kingella*	0.705	0.001	DT - S	*Kingella*	0.723	< 0.001
PT - S	*Treponema*	0.697	0.001	DT - S	*Abiotrophia*	0.681	0.001
PT - S	*Neisseria*	0.639	0.002	DT - S	*Neisseria*	0.669	0.001
PT - S	*Lautropia*	0.629	0.003	DT - S	*Tannerella*	0.666	0.001
PT - S	*Paludibacter*	0.614	0.004	DT - S	*Dialister*	0.644	0.002
PT - S	*Prevotella*	0.588	0.006	DT - S	*Capnocytophaga*	0.626	0.003
PT - S	*Gemella*	0.586	0.007	DT - S	*Lautropia*	0.605	0.005
PT - S	*Peptostreptococcus*	0.542	0.013	DT - S	*Peptococcus*	0.595	0.006
PT - S	*Streptococcus*	0.532	0.016	DT - S	*Propionivibrio*	0.592	0.006
PT - S	*Abiotrophia*	0.531	0.016	DT - S	*Fusobacterium*	0.562	0.010
PT - S	*Anaerovorax*	0.530	0.016	DT - S	*Mycoplasma*	0.544	0.013
PT - S	*Megasphaera*	0.507	0.022	DT - S	*Filifactor*	0.543	0.013
PT - S	*Veillonella*	0.505	0.023	DT - S	*Parvimonas*	0.528	0.017
